# Anti-PD-1 antibodies as a salvage therapy for patients with diffuse large B cell lymphoma who progressed/relapsed after CART19/20 therapy

**DOI:** 10.1186/s13045-021-01120-3

**Published:** 2021-07-05

**Authors:** Chunmeng Wang, Fengxia Shi, Yang Liu, Yajing Zhang, Liang Dong, Xiang Li, Chuan Tong, Yao Wang, Liping Su, Jing Nie, Weidong Han

**Affiliations:** 1 Department of Bio-Therapeutic, the First Medical Centre in Chinese PLA General Hospital, Beijing, China; 2 Department of Hematology, Shanxi Cancer Hospital, Taiyuan, Shanxi China

**Keywords:** DLBCL, Anti-PD-1, CART, Salvage therapy, PD-1/PD-L1

## Abstract

CD19-directed chimeric antigen receptor T cell (CART19) therapy is efficient and approved for relapsed/refractory diffuse large B cell lymphoma (DLBCL). To increase durable antitumor response, we previously designed tandem CART19/20 cells and shown longer progression-free survival. However, a proportion of CART19/20-treated patients will finally progress and require salvage therapies. In this study, we analyzed data from five patients with relapsed/refractory DLBCL who had disease progression or relapse following CART19/20 therapy and then treated with PD-1-blocking antibodies as salvage therapy. Two of five patients acquired complete remissions after anti-PD-1 therapy, including one patient remained ongoing remission for more than 21 months. One patient achieved a partial remission, and the other two had progressive diseases. No ≥ grade 3 treatment-related adverse events or cytokine release syndrome was observed. Immunohistochemistry of tumor specimens revealed higher PD-1/PD-L1 expression in responsive patients with anti-PD-1 therapy as compared to that in non-responders. After anti-PD-1 treatment, circulating T cells were activated in responders, and no significant expansion of CART19/20 cells was detected. Our data suggest that PD-1 blockade therapy can be active in patients with relapsed/refractory DLBCL after failure of CAR T cell therapy who had PD-L1 expression in tumor cells and high PD-1 level in tumor-infiltrated T cells.

**Supplementary Information:**

The online version contains supplementary material available at 10.1186/s13045-021-01120-3.

## To the Editor,

Diffuse large B cell lymphoma (DLBCL) is the most common type of aggressive non-Hodgkin lymphoma (NHL) worldwide, accounting for 30–40% of adult NHL [1]. Although approximately 60–70% of patients are cured with standard frontline therapy, the remaining patients are refractory to frontline therapy or relapse after complete remission [2, 3]. Patients with relapsed/refractory DLBCL respond poorly to other line of chemotherapy, and few patients experience long-term survival [4]. CD19-targeted CAR T cell (CART19) therapy has remarkably improved the outcome of aggressive B cell lymphoma, and 52–83% of patients had a response including 40–58% achieving a complete remission, with a median progression-free survival of 5.9 months [5, 6]. In order to potentiate the long-term efficiency of CART therapy, we previously designed and reported that tandem CART19/20 had a robust antitumor activity, and 64% of patients resulted in durable response for more than one year [7]. Despite the encouraging results, a part of patients eventually experienced disease progression or relapse after CART19/20 therapy. Effective treatment strategies for those patients post-CART19 or CART19/20 failure are imperative but limited.

Programmed cell death-1 (PD-1) is a key immune checkpoint that suppresses T cell-mediated immune response. Emerging evidence has suggested aberrant PD-L1 expression on tumor cells elicited inhibitory signals, caused CAR T cell exhaustion and impaired tumor cell killing, regarding as one mechanism in the setting of relapses after CART therapy [8]. The combination of CART therapy and PD-1 blockade therapy has been conducted in preclinical models and clinical trials, in order to escalate CAR T cell function and enhance the antitumor efficacy [9, 10]. Moreover, engineered CAR T cells producing PD-1-neutralizing scFv displayed improved survival in mouse solid tumor models [11]. However, the effect of PD-1 blockade therapy in patients with B cell lymphoma who failed CAR T cell therapy was not clear. Here, we reported the efficacy and biological characteristics of five DLBCL patients who received PD-1-blocking antibody as a salvage treatment after failure of CART19/20 cell infusion.

Between May 1 and September 21, 2019, five patients with relapsed/refractory DLBCL and recurrent/progressive lymphoma after tandem CART19/20 (TanCAR7 T cells) therapy (NCT03097770) [7] were enrolled. The retrospective study was approved by the Ethics Committee of Chinese PLA General Hospital and conducted in accordance with principles of the Declaration of Helsinki. Informed consent was obtained from all patients. Patients were at a median age of 41 years (range 38–55 years) and had primary refractory (n = 3) or relapsed (n = 2) non-germinal center B cell DLBCL, and three patients had extranodal lesions. All patients had received three or more previous regimens (range 3 to 9), and CART19/20 therapy was the most recent treatment with a median progression-free survival (PFS) of 5 months (Table 1). After failing CART therapy, patients received PD-1-blocking antibody (sintilimab or camrelizumab) at 200 mg every 2 weeks as a salvage treatment. Treatment continued until disease progression or unacceptable toxicity occurred. Patients with sustained CRs received consolidate treatment per 4 weeks. As of May 1, 2021, the median follow-up was 21.8 months, one patient remained on treatment, and other four discontinued therapy because of disease progression.

**Table 1:** Baseline clinical characteristics and post-anti-PD-1 outcomes

Patient no.	1	2	3	4	5
Age (years)	54	40	41	38	35
Sex	M	F	F	M	F
ECOG performance status	0	2	1	0	2
Diagnosis/stage	DLBCL/IV	DLBCL/IV	DLBCL/III	DLBCL/IV	DLBCL/III
Disease status^1^	Relapsed	Primary refractory	Primary refractory	Relapsed	Primary refractory
Target lesion	Bilateral parotid nodule	Bone marrow, spleen, lymph nodes (9 regions)	Lymph nodes (2 regions)	Bone, lymph node	Lymph node
Prior system regimens	RCHOP × 5, RDICE × 3, RGEMOX × 9, RMTX × 2, RAD × 6, BEACOPP × 2	RCHOP × 8, DHAP × 4, CART19	CHOPE × 4, RDICE × 6, RDHAP × 4, IR × 2	RCHOPE × 2, ABVD × 3, DICE × 4, ESHAP × 7, BEAM × 2, R2 × 6	REPOCH × 8, GEMOX × 2
Prior RT	Yes	No	No	No	No
Prior ASCT	Yes	No	No	No	No
Prior CD19/CD20 CAR-T	Yes	Yes	Yes	Yes	Yes
Best response	CR	CR	PR	CR	PD
Final response	PD	PD	PD	PD	PD
PFS (months)	6	6	5	3	3
Percentage of PD-L1^+^ expression^2^	0	0	30%	70%	0
Percentage of PD-L1^+^ expression^3^	30%	0	40%	80%	0
Percentage of PD-1^+^ expression^2^	0	0	20%	0	0
Percentage of PD-1^+^ expression^3^	80%	5%	60%	60%	0
Anti-PD-1 therapy					
Antibody type (treatment cycle)	Sintilimab (12)	Camrelizumab (1)	Sintilimab (5)	Camrelizumab (24)	Sintilimab (2)
Best response	CR	PD	PR	CR	PD
Final response	PD	PD	PD	CR	PD
PFS (months)	10.1	0.63	3.3	21.8	1
OS (months)	NE	16.2	NE	NE	15.6
TrAE (grade)	Increased transaminase (1)	None	Fever (2), leukocytopenia (2)	Fever (1), rash (1)	None

ASCT, autologous stem-cell transplantation; ABVD, doxorubicin, bleomycin, vindesine, dacarbazine; BEACOPP, bleomycin, etoposide, doxorubicin, cyclophosphamide, vindesine, procarbazine, prednisone; BEAM, carmustine, etoposide, cytarabine, melphalan; CHOPE, cyclophosphamide, vindesine, doxorubicin, etoposide, prednisone; DHAP, cisplatin, cytarabine, dexamethasone; ESHAP, rituximab, etoposide, vindesine, doxorubicin, cyclophosphamide, prednisone; GEMOX, gemcitabine, oxaliplatin; RCHOP, rituximab, cyclophosphamide, vindesine, doxorubicin, prednisone; RMTX, rituximab, methotrexate; RAD, rituximab, cytosine arabinoside, dexamethasone; RDHAP, rituximab, cisplatin, cytarabine, dexamethasone; RDICE, rituximab, ifosfamide, cisplatin, etoposide, dexamethasone; REPOCH, rituximab, etoposide, vindesine, doxorubicin, cyclophosphamide, prednisone; RGEMOX, rituximab, gemcitabine, oxaliplatin; RFC, rituximab, fludarabine, cyclophosphamide; R2, rituximab combined with lenalidomide; DLBCL, diffuse large B cell lymphoma; ECOG, Eastern Cooperative Oncology Group; GCB, germinal center B cell; PFS, progression-free survival; TrAE, treatment-related adverse events

^1^Primary refractory, non-response or relapse within 3 months of frontline therapy

^2^These data were collected prior to CART19/20 therapy

^3^These data were collected after failure of CART19/20 therapy

Overall, three of five patients experienced treatment-related adverse events, including fever in two patients, rash, leukocytopenia and increased transaminase in one patient, respectively (Table 1). No ≥ 3 grade toxicities or treatment-related deaths occurred. Besides, cytokine release syndrome (CRS) was not observed after anti-PD-1 antibody therapy in all patients, although they had received infusion of CART19/20 cells within six months.

**Fig. 1:**
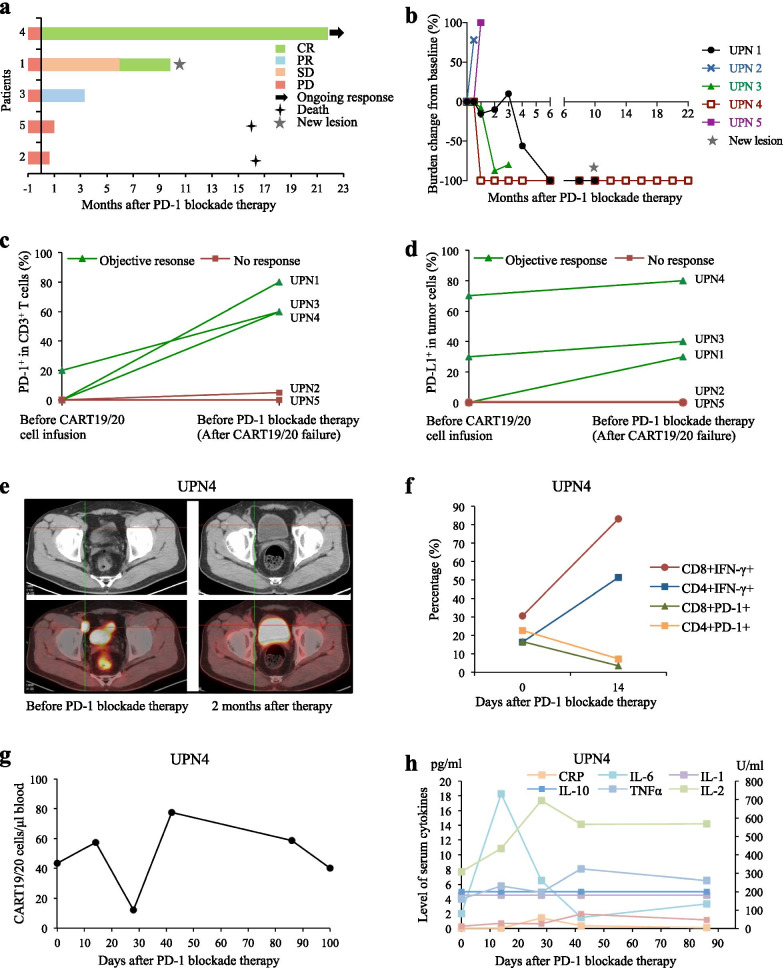
Clinical response to anti-PD-1 therapy and biological biomarker detection. **a** Anti-PD-1 treatment response of each patient and duration of response following anti-PD-1 salvage therapy. UPN1 had 100% decrease in baseline tumor burden and another new lesion occurred, and evaluated as disease progression after 10 months. CR, complete response; PR, partial response; SD, stable disease; PD, progressive disease. **b** Percentage change of tumor burden after anti-PD-1 therapy compared to baseline level prior to anti-PD-1. **c**, **d** Percentage of PD-1^+^CD3^+^ cells in tumor-infiltrated CD3^+^ cells (**c**) or PD-L1^+^ cells in CD19 and CD20 tumor cells (**d**) from all five patients before CART19/20 cell infusion and after disease progression or relapse post-CART therapy. Responses to anti-PD-1 salvage therapy were shown. **e** PET/CT imaging for UPN4 before anti-PD-1 and 2 months after anti-PD-1 therapy. **f** Percentage of IFN-γ^+^CD8^+^ cells, IFN-γ^+^CD4^+^ cells, PD-1^+^CD8^+^ cells and PD-1^+^CD4^+^ cells in peripheral blood prior to anti-PD-1 and 14 days after anti-PD-1 treatment in UPN4 by flow cytometry. **g** The absolute number of CART19/20 cells in UPN4 by PCR. **h** Serum cytokine levels in UPN4 following PD-1 blockade therapy. UPN, unique patient number

The antitumor response was evaluated using FDG-PET-CT at baseline and every 2 to 3 months, based on the Revised Response Criteria for Malignant Lymphomas (Lugano classification). As shown in Table 1 and Fig. 1a, b, three of the five patients achieved objective responses after PD-1 blockade therapy, including two CRs and one PR; the other two patients had progressive diseases and died on 15.6–16.2 months. UPN4 maintained durable CR till data cutoff date with response duration of 21.3 month, and PFS was 21.8 months. The baseline tumor in UPN1 was completely eliminated, while another new lesion occurred and evaluated as disease progression after 10 months (Additional file 1: Fig. S1).

As detected by immunohistochemical staining, tumor cells from all five patients expressed CD19 and CD20 both prior to and after failure of CART19/20 therapy, with more CD3^+^ T cells in tumor microenvironment at relapse (Additional file 1: Fig. S2). The H-scores for PD-1 and PD-L1 are listed in Additional file 1: Table S1. Interestingly, we observed significantly higher PD-1 expression in CD3^+^ T cells and more PD-L1^+^ tumor cells in patients responding to anti-PD-1 treatment (UPN1, 3 and 4) compared to those without a response (UPN2 and 5; Fig. 1c, d, Table 1). Especially for UPN4 whose tumor cells strongly expressed PD-L1, PD-1 expression in tumor-infiltrated CD3^+^ cells was not detectable prior to CART19/20 cell infusion and increased to 60% when tumor relapsed; after two-month anti-PD-1 camrelizumab salvage treatment, he obtained a CR (Fig. 1e). After camrelizumab treatment, peripheral T cells were activated as increased percentage of IFN-γ-expressing T cells and decreased PD-1 level (Fig. 1f). Serum cytokine IL-6 and IL-2 were elevated within 4 weeks, while only a tiny expansion of circulating CART19/20 cells occurred on 6 weeks (Fig. 1g, h). Increased levels of serum cytokines and T cell activation were also detected in UPN3 (Additional file 1: Fig. S3). Based on our results, at treatment failure of CART therapy, tumor PD-L1 expression was weakly upregulated, and tumor-infiltrated T cells in some patients were highly exhausted and might have responsiveness to PD-1 blockade therapy.

Till now, effective salvage treatments for patients with aggressive DLBCL who experience relapsed/refractory lymphoma after CART19 therapy are urgently needed. Infusion of different CART cells, such as CD28-based CART19, CD19-PD-1/CD28-CAR-T, or a second CART19 infusion had been used, achieving an objective response of 33–52% [12–14]. Elise A. Chong et al. reported a significant antitumor response of pembrolizumab in one case with DLBCL and progressive lymphoma after CART19, with expansion of CART19 cells following pembrolizumab [15]. In the present study, among five patients after failing CART19/20 therapy, three had objective responses (2 CRs and 1 PR) with a salvage therapy of anti-PD-1 antibodies, and no serious toxicities experienced.

Loss of tumor antigens and emergence of an immunosuppressive tumor microenvironment contributed to the adaptive resistance to CART immunotherapy [8]. In this study, none of the five patients lost tumor antigen expression at disease recurrence or progression post-CART19/20 therapy, while PD-1 expression in CD3^+^ T cells was dramatically elevated in three patients. Notably, these three patients acquired a response to anti-PD-1 and UPN4 whose tumor cells had the highest PD-L1 expression (80%) achieved a long-lasting remission. Moreover, the antitumor effect of anti-PD-1 salvage therapy might not be relied on enhancing the efficacy of CART cells, since we did not observe significant expansion of CART19/20 cells and related elevation of serum cytokines. However, after CART therapy these three patients’ lymphomas changed into “hot tumor” and responded to anti-PD-1. Thus, PD-1/PD-L1 pathway might be crucial in controlling the effectiveness and overcoming the resistance to CART therapy.

Together, our data suggest that PD-L1 expression in tumor cells and high PD-1 level in T cells might be potential biomarkers to predict the outcome of anti-PD-1 antibodies in relapsed/refractory DLBCL and PD-1 blockade therapy is recommended for patients in this setting after failure of CART therapy. To further increase clinical response, we are planning to conduct a phase I/II clinical trial of anti-PD-1-based combination therapy in patients with DLBCL failing to response to CART therapy.

## Supplementary Information

**Additional file 1**. **Figure S1.** PET/CT imaging for UPN1. **Figure S2.** Immunohistochemistry in tumor samples from all five patients. **Figure S3.** Biological biomarker detection from blood samples. **Table S1.** H-scores for PD-1 and PD-L1 in patients before CART19/20 therapy and after failure of CART19/20.

## Data Availability

Additional data are provided in the data supplement available online. Individual participant data will be shared by the corresponding author on reasonable request.
