# Correction to: Anti-PD-1 antibodies as a salvage therapy for patients with diffuse large B cell lymphoma who progressed/relapsed after CART19/20 therapy

**DOI:** 10.1186/s13045-021-01154-7

**Published:** 2021-09-23

**Authors:** Chunmeng Wang, Fengxia Shi, Yang Liu, Yajing Zhang, Liang Dong, Xiang Li, Chuan Tong, Yao Wang, Liping Su, Jing Nie, Weidong Han

**Affiliations:** 1 Department of Bio-Therapeutic, The First Medical Centre in Chinese PLA General Hospital, Beijing, China; 2 Department of Hematology, Shanxi Cancer Hospital, Taiyuan, Shanxi China

## Correction to: J Hematol Oncol (2021) 14:106 10.1186/s13045-021-01120-3

The original article [1] contained a minor error in the Additional file which has since been corrected.
