# Supplementary material for: Anti-PD-1 antibodies as a salvage therapy for patients with diffuse large B cell lymphoma who progressed/relapsed after CART19/20 therapy
Source: J Hematol Oncol. 2021 Jul 5;14:106. doi: 10.1186/s13045-021-01120-3 (PMC8259370; doi:10.1186/s13045-021-01120-3)
Supplement: Supplementary file 1 — Additional file 1. Figure S1. PET/CT imaging for UPN1. Figure S2. Immunohistochemistry in tumor samples from all five patients. Figure S3. Biological biomarker detection from blood samples. Table S1. H-scores for PD-1 and PD-L1 in patients before CART19/20 therapy and after failure of CART19/20. [file 13045_2021_1120_MOESM1_ESM.docx]

**Additional file 1**

**Anti-PD-1 antibodies as a salvage therapy for patients with diffuse large B-cell lymphoma who progressed/relapsed after CART19/20 therapy**

Chunmeng Wang, Fengxia Shi, Yang Liu, Yajing Zhang, Liang Dong, Xiang Li, Chuan Tong, Yao Wang, Liping Su, Jing Nie, Weidong Han

**Contents**

**Supplementary Methods** 3

Flow cytometry analysis 3

Immunohistochemical staining and analysis 3

**Supplementary Figures** 4

Figure S1. PET/CT imaging for UPN1. 4

Figure S2. Immunohistochemistry in tumor samples from all five patients. 5

Figure S3. Biological biomarker detection from blood samples. 7

**Supplementary Tables** 8

Table S1. H-scores for PD-1 and PD-L1 in patients before CART19/20 therapy and after failure of CART19/20. 8

# Supplementary Methods

## Flow cytometry analysis

The peripheral blood was collected in sodium heparin anticoagulant vacutainer tubes. After red blood cell lysis and washing, cells were stained with the indicated antibodies according to the manufacture’s instruments, and detected on a BD FACSCalibur flow cytometer (BD Biosciences), a minimum of 10,000 CD3^+^ lymphocytes were collected. Following antibodies were purchased from BD Biosciences: anti-CD3-PerCP (347344), anti-CD8-APC (340584), anti-IFN-γ-FITC (554700), anti-PD-1-PE (560795) and isotype-matched antibodies.

## Immunohistochemical staining and analysis

On tumor samples, we performed the immunohistochemical staining of CD19 (clone UMAB103, ZM-0038) and CD20 (clone L26, PA0200) to indicate the malignant NHL cells, CD3 (cone LN10, ZM-0417) staining as T-cells, and detect PD-L1 (clone ZR3, GT228004) and PD-1 (clone UMAB199, ZM-0381) expression. The percentage of tumor (CD19^+^ and CD20^+^) cells with PD-L1-positive staining was calculated, as analyzed on more than 50 tumor cells. The percentage of T-cells (CD3^+^) with PD-1-positive staining was calculated, as analyzed on more than 50 T-cells. PD-1 and PD-L1 expressions were scored using histoscore (H-score, range 0-300), which was calculated by multiplying the percentage of positively stained cells, with staining intensity as 0 (negative), 1+ (weak positive), 2+ (positive) and 3+ (high positive).

# Supplementary Figures

## Figure S1. PET/CT imaging for UPN1.

PET/CT imaging for UPN1 before PD-1 blockade therapy and the indicated months after PD-1 blockade therapy were shown. The red arrow indicated the baseline tumor lesion and the blue arrow indicated another new lesion.

## Figure S2. Immunohistochemistry in tumor samples from all five patients.

Expressions of CD19, CD20, CD3, PD-1 and PD-L1 in tumor biopsies prior to tandem CART19/20 infusion and prior to PD-1 blockade therapy (after failure of CART19/20 infusion) were detected by immunohistochemical staining. These images are at ✕ 400 magnification.

## Figure S3. Biological biomarker detection from blood samples.

**a** The absolute number of CART19/20 cells in UPN2, UPN3 and UPN5 by PCR. **b** Serum cytokine levels in UPN2, UPN3 and UPN5 following PD-1 blockade therapy. **c** Percentage of IFN-γ^+^CD8^+^ cells, IFN-γ^+^CD4^+^ cells, PD-1^+^CD8^+^ cells and PD-1^+^CD4^+^ cells in peripheral blood prior to anti-PD-1 and 14 days after anti-PD-1 treatment in UPN3 by flow cytometry.

# Supplementary Tables

## Table S1. H-scores for PD-1 and PD-L1 in patients before CART19/20 therapy and after failure of CART19/20.

| Patient No. | 1 | 2 | 3 | 4 | 5 |
| --- | --- | --- | --- | --- | --- |
| PD-L1 before CART19/20 therapy | 0 | 0 | 90 | 210 | 0 |
| PD-L1 after failure of CART19/20 | 90 | 0 | 120 | 240 | 0 |
| PD-1 before CART19/20 therapy | 0 | 0 | 60 | 0 | 0 |
| PD-1 after failure of CART19/20 | 240 | 15 | 180 | 180 | 0 |
